# Tumor lysate-based vaccines: on the road to immunotherapy for gallbladder cancer

**DOI:** 10.1007/s00262-018-2157-5

**Published:** 2018-03-29

**Authors:** Daniel Rojas-Sepúlveda, Andrés Tittarelli, María Alejandra Gleisner, Ignacio Ávalos, Cristián Pereda, Iván Gallegos, Fermín Eduardo González, Mercedes Natalia López, Jean Michel Butte, Juan Carlos Roa, Paula Fluxá, Flavio Salazar-Onfray

**Affiliations:** 1 Disciplinary Program of Immunology, Institute of Biomedical Sciences, Faculty of Medicine, Universidad de Chile, Independencia 1027, building H, Third floor, 8380453 Santiago, Chile; 2 Millennium Institute on Immunology and Immunotherapy, Universidad de Chile, 8380453 Santiago, Chile; 3 Faculty of Science, Universidad San Sebastián, Lota 2465, 7510157 Santiago, Chile; 4 Department of Conservative Dentistry, Faculty of Dentistry, Universidad de Chile, 8380492 Santiago, Chile; 5 Pathological Anatomy Service, Clinic Hospital, Universidad de Chile, 8380456 Santiago, Chile; 6 Department of Surgery, Fundación Arturo López Pérez, Institute of Oncology, 7500921 Santiago, Chile; 7 Department of Pathology, School of Medicine, Pontificia Universidad Católica de Chile, 8330023 Santiago, Chile; 8 Center for Investigation in Translational Oncology (CITO), Advanced Center for Chronic Diseases (ACCDiS), School of Medicine, Pontificia Universidad Católica de Chile, 8330023 Santiago, Chile

**Keywords:** Melanoma, Gallbladder cancer, Dendritic cells, Tumor lysates, Immunotherapy, CITIM 2017

## Abstract

Immunotherapy based on checkpoint blockers has proven survival benefits in patients with melanoma and other malignancies. Nevertheless, a significant proportion of treated patients remains refractory, suggesting that in combination with active immunizations, such as cancer vaccines, they could be helpful to improve response rates. During the last decade, we have used dendritic cell (DC) based vaccines where DCs loaded with an allogeneic heat-conditioned melanoma cell lysate were tested in a series of clinical trials. In these studies, 60% of stage IV melanoma DC-treated patients showed immunological responses correlating with improved survival. Further studies showed that an essential part of the clinical efficacy was associated with the use of conditioned lysates. Gallbladder cancer (GBC) is a high-incidence malignancy in South America. Here, we evaluated the feasibility of producing effective DCs using heat-conditioned cell lysates derived from gallbladder cancer cell lines (GBCCL). By characterizing nine different GBCCLs and several fresh tumor tissues, we found that they expressed some tumor-associated antigens such as CEA, MUC-1, CA19-9, Erb2, Survivin, and several carcinoembryonic antigens. Moreover, heat-shock treatment of GBCCLs induced calreticulin translocation and release of HMGB1 and ATP, both known to act as danger signals. Monocytes stimulated with combinations of conditioned lysates exhibited a potent increase of DC-maturation markers. Furthermore, conditioned lysate-matured DCs were capable of strongly inducing CD4^+^ and CD8^+^ T cell activation, in both allogeneic and autologous cell co-cultures. Finally, in vitro stimulated CD8^+^ T cells recognize HLA-matched GBCCLs. In summary, GBC cell lysate-loaded DCs may be considered for future immunotherapy approaches.

## Introduction

The recent use of immune-checkpoint blocker antibodies has demonstrated durable clinical benefits in patients with melanoma, lung cancer and other solid tumors [3–9]. Despite this relevant clinical performance, a high percentage of treated patients remains refractory, strongly suggesting that the combination with active immunizations may be useful to improve the response rates of those patients. In this context, cancer vaccines, particularly dendritic cell (DC)-based vaccines, can be used as complementary treatments in cancer patients. Optimal delivery of a wide-ranging pool of tumor-associated antigens (TAAs) and the use of adequate adjuvants are shown to be crucial for vaccine success [10]. During the last decade, we have been able to produce therapeutic DCs using an allogeneic heat-conditioned melanoma cell lysate named TRIMEL. Sixty percent of advanced melanoma patients treated with these DCs showed a delayed type hypersensitivity reaction against TRIMEL, which correlated with a threefold prolonged survival [11]. This strategy provides a reproducible pool of almost all the potential melanoma-associated antigens, suitable for use in a wide range of patients independent of their major histocompatibility complex (MHC) haplotypes or the availability of autologous tumor tissue [12]. Moreover, we previously showed that TRIMEL contains some heat shock-induced damage-associated molecular patterns (DAMPs), such as high mobility group box-1 (HMGB1) and calreticulin (eCRT), which mediate an optimal maturation, activation and antigen cross-presentation of the monocyte-derived DCs, and thus enable them to activate antigen-specific T cells [13]. However, the development of an optimal allogeneic tumor cell lysate preparation for different tumor types is crucial to expand the use of these approaches for different cancers.

Gallbladder cancer (GBC) is the most common cancer of the biliary tree. Although GBC is infrequent in developed countries [14], in South America and particularly in Chile, this tumor constitutes a major health problem [14–17]. The underlying causes for the high risk of GBC in these areas are unclear, but several important risk factors probably contribute, including chronic inflammation caused by gallstones, high obesity rates and genetic susceptibility in women of indigenous *Mapuche* ancestry, in which the incidence increases to 27.3 cases per 100,000 [14, 16, 17].

Early detection and diagnosis of GBC is complicated because the clinical symptoms are manifested in advanced stages. The average survival time for patients with advanced, non-resectable GBC varies from 4 to 14 months [17, 18]. The most effective treatment for this type of cancer is surgical removal of the primary tumor and areas of local extension. Unfortunately, less than 10% of patients have resectable tumors, and nearly 50% of them present metastasis at the time of diagnosis [19]. Even with surgery, most of the GBC patients progress to a metastatic stage, highlighting the need for novel adjuvant therapies, such as immunotherapy.

The purpose of this study was to investigate the immunogenicity of several combinations of tumor lysates derived from different GBC cell lines (GBCCL) and their effect on monocyte differentiation and activation to DCs and their capacity to induce an in vitro T cell-mediated anti-GBC response. In this respect, a major requirement for the potential clinical effectiveness of GBC lysate-loaded DCs is to investigate the presence of shared TAAs in GBCCL and in fresh tumor tissues. Our results suggest that human DCs matured with specific GBCCL heat shock-conditioned lysates are capable of inducing specific T cells activation against this tumor and can be considered for the development of future immunotherapeutic approaches for GBC patients.

## Materials and methods

### Cell lines and cell lysates

GBCCL GBd1 (CVCL_H705), G415 (CVCL_8198), OCUG-1 (CVCL_3083), NOZ (CVCL_3079), TGBC-1TKB (CVCL_1769; hereafter 1TKB), TGBC-2TKB (CVCL_3339; hereafter 2TKB), TGBC-14TKB (CVCL_3340; hereafter 14TKB) and TGBC-24TKB (CVCL_1770; hereafter 24TKB) were provided by Juan Carlos Roa (Department of Pathology, Pontificia Universidad Católica de Chile, Santiago, Chile). The GBCCL CAVE was established in our lab from a primary adenocarcinoma GBC tumor sample from a Chilean patient. NOZ, GBd1 and G415 cells were grown in RPMI 1640 culture medium (Corning, NY, USA), whereas OCUG-1, 1TKB, 2TKB, 14TKB, 24TKB and CAVE were grown in DMEM culture medium (Corning, NY, USA). Culture media were supplemented with 10% fetal bovine serum (FBS), 10 U/mL penicillin and 10 mg/mL streptomycin (Corning, NY, USA). Cells were maintained at 37 °C under 5% CO_2_ and 95% relative humidity.

Cell lysates were produced as previously described [13]. Briefly, for individual GBCCL lysates, 4 × 10^6^ cells/mL were heat shocked at 42 °C for 1 h, incubated for 2 h at 37 °C and then lysed. For GBCCL combined lysates, cells were mixed in equal amounts to achieve a final concentration of 4 × 10^6^ cells/mL, and heat shocked as described before. The mixed cell lysates evaluated were made as follows: M1 (24TKB + GBd1 + G415); M2 (2TKB + 24TKB + GBd1); M3 (1TKB + 2TKB + 24TKB); M4 (OCUG1 + GBd1 + G415); M5 (2TKB + G415 + OCUG1); M6 (NOZ + OCUG 1 + G415); M7 (1TKB + 14TKB + 24TKB); and M8 (24TKB + OCUG1 + G415).

### Antibodies

Monoclonal antibodies (mAbs) against human carcinoembryonic antigen (CEA; clone COL-1), erbB2 (clone 3B5), and survivin (clone 8E2) were purchased from Thermo Fisher Scientific (Waltham, Massachusetts, USA). mAbs against human mucin-1 (MUC-1; clone HMFG1), cancer antigen 19-9 (CA19-9; clone SPM110) and calreticulin (clone FMC 75) were purchased from Abcam (Cambridge, USA). mAbs against human CD3 eFluor450 (clone SK7), human leukocyte antigen (HLA)-DR APC eFluor780 (clone LN3), CD83 PE Cy7 (clone HB15e), CD25 PerCP Cy5.5 (clone BC96), CD69 PE (clone FN50) and interleukin (IL)-4 PE Cy7 (clone 8D4-8) were purchased from eBioscience (San Diego, CA, USA). mAbs against human CD8 PE Cy7 (clone SK1), C-C chemokine receptor type 7 (CCR7) PE (clone G043H7), CD4 APC Cy7 (clone RPA-T4), tumor necrosis factor (TNF)-α PerCP (clone Mab11) and interferon (IFN)-γ AlexaFluor 647 (clone 4S.B3) were purchased from BioLegend (San Diego, CA, USA). Polyclonal goat anti-mouse IgG antibody was purchased from eBioscience. mAbs against human HLA-ABC (clone G46-2.6), CD80 BV421 (clone L307.4), CD86 BB515 (clone 2331), C-X-C motif chemokine receptor (CXCR)3 APC (clone 1C6/CXCR3) and CXCR4 APC (clone 12G5) were purchased from BD Pharmingen (San Diego, CA, USA).

### Flow cytometry

The surface expression of MUC-1, erbB2, survivin, CA19-9, CEA, and eCRT was analyzed by flow cytometry. Intracellular staining was performed with the Foxp3/Transcription Factor Fixation/Permeabilization Concentrate and Diluent kit (eBioscience). Live/dead kit (Thermo Fisher) was used for live/dead cell discrimination. Flow cytometry was conducted on a FACSVerse flow cytometer (BD Biosciences) and data analysis was performed using the FlowJo software (Tree Star, Inc., Ashland, OR, USA).

### Reverse transcription polymerase chain reaction (RT-PCR)

Total RNA was extracted from cells using TriPure reagent (Roche) and used to determine the expression and relative level of the Melanoma-associated antigen (MAGE), G antigen (GAGE) and B melanoma antigen (BAGE) in GBCCL. cDNAs were synthesized with M-MLV Reverse Transcriptase (Life Technologies). PCR was performed using cDNA template in the MasterCycler (Eppendorf), according to the manufacturer’s instructions. The sequences of the used primers are available under request.

### Immunohistochemistry

Sections of 3 µm thickness from paraffin-embedded GBC tissues were mounted on slides, rehydrated and antigen retrieval was performed by heat in Tris–EDTA pH 9.0 or citrate buffer pH 6.0 depending on the Ab used. Primary Abs were used according to manufacturer’s instructions (CEA dilution 1:200, clone COL-1, Thermo Scientific; MUC-1 dilution 1:200, clone HMFG1, Abcam; erbB2 dilution 1:200, clone 3B5, Thermo Scientific; CA19-9 dilution 1:50, clone SPM110, Abcam; and survivin dilution 1:50, clone 8E2, Thermo Scientific). The slides were incubated with primary Abs in a moist chamber overnight at 4 °C. After incubation with primary Abs, slides were washed with TBS before incubation with labeled secondary Abs for 1 h at 4 °C. Sections were subsequently incubated with ABC solution for 30 min (ABC Vectastain Kit Elite PK6200, Vector Laboratories), washed with three changes of TBS, incubated with Dako-Chromogen solution and washed with deionized water. Background staining was performed with Mayer’s hematoxylin, sections were dehydrated through ascending alcohols to xylene and mounted. Negative control slides omitting the primary Ab were included in all batches. An expert pathologist evaluated the expressions of CEA, MUC-1, c-erbB2, CA19-9 and survivin in GBC tissues.

### Enzyme-linked immunosorbent assay (ELISA)

The concentration of HMGB1 in 100 µL of supernatants from control and heat shocked GBCCL (4 × 10^6^ cells/mL) were measured by ELISA using a specific HMGB1 ELISA kit according to the manufacturer’s instructions (Cloud-Clone Corp.). 450 nm optical densities were measured in a Sunrise absorbance reader (Tecan).

### ATP determination

The concentration of ATP in supernatants from control and heat shocked GBCCL (4 × 10^6^ cells/mL) was measured by the Luciferase-Based ATP Determination Kit (Life Technologies) according to the manufacturer’s instructions. Luminescence was measured in a TopCount luminescence counter (PerkinElmer).

### DC generation

Adherent monocytes isolated from peripheral blood mononuclear cells (PBMC) of healthy donors from the Centro Metropolitano de Sangre y Tejidos, Hospital Metropolitano (Santiago, Chile) were cultured in serum-free AIM-V medium (Invitrogen) for 22 h with 500 U/mL recombinant human IL-4 (rhIL-4; US-Biological) and 800 U/mL recombinant human granulocyte–macrophage colony-stimulating factor (rhGM-CSF; Sheering Plough) and then stimulated for 24 h with 100 µg/mL of GBCCL lysates, TRIMEL (TRIMEL-DCs) or with medium [activated monocytes (AM)] as previously described [20].

### DC/T cell co-cultures

For allogeneic cell co-cultures, CD3^+^ T cells from healthy donors were sorted with a FACSAria II sorter (BD Biosciences) and co-cultured for 5 days with TRIMEL-DCs or DCs matured with GBCCL lysates at a 20:1 ratio in RPMI 1640 medium supplemented with 10% FBS and 150 UI/mL rhIL-2 (Proleukin). For autologous co-cultures, sorted CD3^+^ T cells from HLA-A2^+^ healthy donors were co-cultured with AM, TRIMEL-DCs or DCs matured with the M2 lysate (M2-DCs) for 14 days at a 10:1 ratio in RPMI 1640 medium supplemented with 10% FBS and 150 UI/mL rhIL-2. T cells were re-stimulated at day 7 with freshly prepared DCs maintaining the cell:cell ratio. Surface expression of CD25, CD69, CXCR3 and CXCR4 was analyzed in CD4^+^ and CD8^+^ T cells by flow cytometry. For intracellular IFN-γ, TNF-α and IL-4 staining, 1 × 10^6^ T cells were cultured for 4 h at 37 °C in RPMI 1640 medium with 10% FBS containing 1 µg/mL ionomycin, 0.15 µM phorbol myristate acetate (PMA), and 3 µg/mL brefeldin A. T cell proliferation was studied using carboxyfluorescein succinimidyl ester (CFSE) dilution analysis.

### IFN-γ ELISpot

Autologous CD8^+^ T cells activated with AM, TRIMEL-DCs or M2-DCs were sorted and co-cultured with 1 × 10^4^ target cells: HLA-A2^+^ GBCCL (GBd1, TGBC-2TKB, CAVE), HLA-A2^+^ melanoma cell line (Mel1) or K562 for 16 h at different effector/target ratios. IFN-γ release was tested by an ELISpot assay according to the manufacturer’s instructions (ELISPOT Ready-SET-Go, eBioscience) as previously described [20].

### Statistical analysis

Statistical analysis was achieved using GraphPad Prism software version 6.0 (GraphPad Software, San Diego, CA, USA). Student’s *t* test was used to determine differences between treatments and results are presented as mean ± standard deviation (SD). *p* values < 0.05 were considered significant.

## Results

### GBCCL express relevant tumor-associated antigens present in GBC tissues

**Fig. 1:**
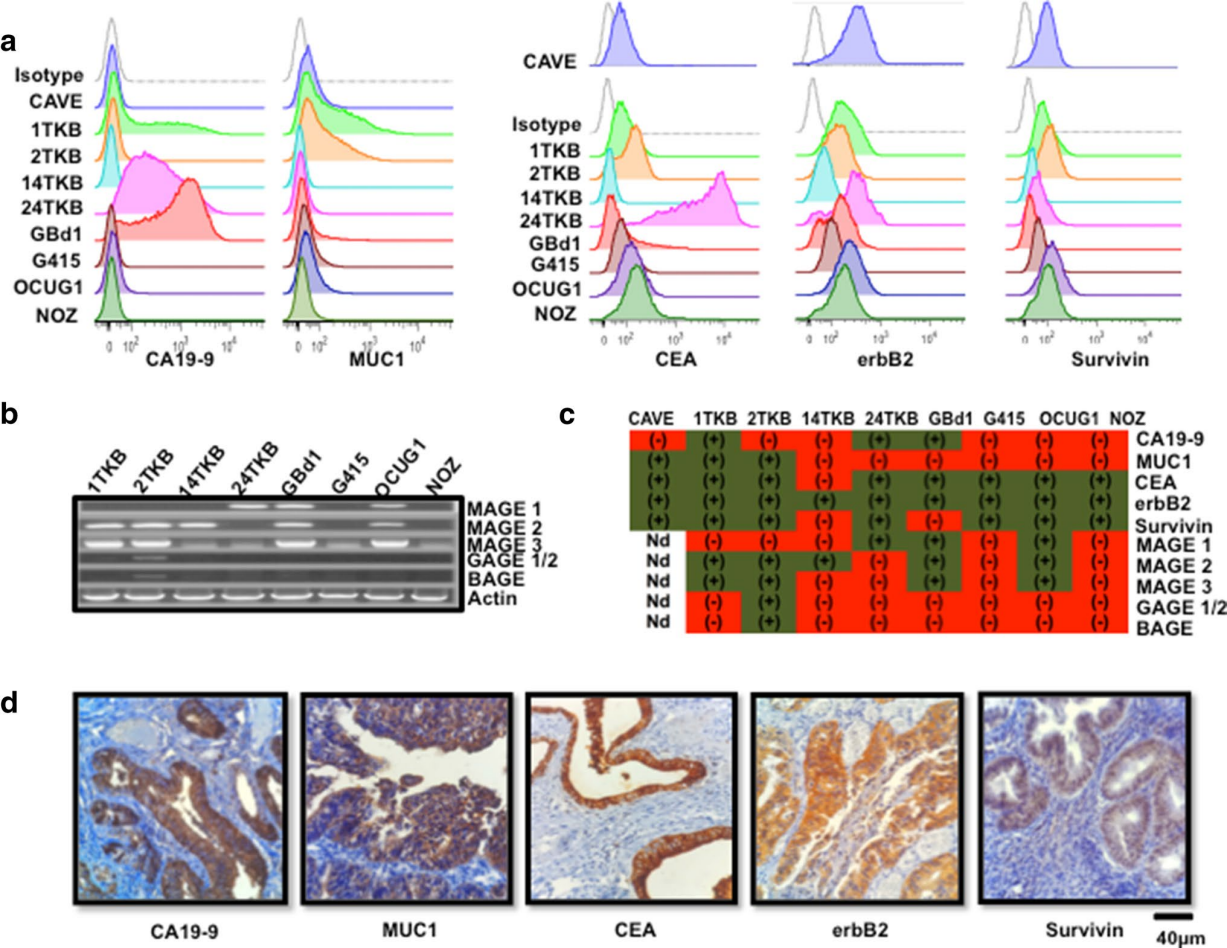
Tumor associated antigen expression in GBCCL and GBC fresh tumor samples. **a** Representative histograms for CA19-9, MUC-1, CEA, erbB2 and survivin expression in GBCCL evaluated by flow cytometry. Grey histograms indicate isotype control staining. **b** mRNA expression profiles for MAGE 1, 2, 3, GAGE 1/2 and BAGE in the GBCCL analyzed by RT-PCR. Actin was used as a housekeeping gene control. **c** Summary of tumor associated antigen expression in GBCCL. Green and red refers to positive or negative expression, respectively. *ND* not determined. **d** Representative photomicrographs of immunohistochemical staining for CA19-9, MUC-1, CEA, erbB2 and survivin in paraffin-embedded tumor biopsies obtained from Chilean GBC patients (scale bar, 40 µm)

To select a GBCCL suitable for the production of cell lysates as a source of multiple tumor antigens, the levels of expression of 10 of the most common and relevant TAAs (survivin, MUC-1, CEA, erbB2, CA19-9, MAGE-1, MAGE- 2, MAGE-3, GAGE-1/2 and BAGE) were determined in eight publicly available GBCCL (GBd1, G415, OCUG-1, NOZ, 1TKB, 2TKB, 14TKB and 24TKB) and in one GBCCL established in our lab (CAVE). The protein levels of survivin, MUC-1, CEA, erbB2 and CA19-9 were determined by flow cytometry, whereas the expression of MAGEs, GAGEs and BAGE was evaluated at the RNA level by RT-PCR. The nine GBCCL showed diverse levels and patterns of antigen expression and none of them expressed all ten antigens, but all expressed at least two of them (Fig. 1a–c). The expression of erbB2 was detected in all the cell lines analyzed, whereas the 2TKB cells expressed only the antigens GAGE1/2 and BAGE. The cell lines with the broader pattern of antigen expression were 2TKB and 1TKB, which express 8 and 7 of the 10 antigens, respectively (Fig. 1c). Additionally, survivin, MUC-1, CEA, erbB2 and CA19-9 antigens were also detected in a significant number of tumor samples from GBC patients (Fig. 1d), suggesting that these were suitable antigen targets for immunotherapy approaches.

### Heat shock induces the production of DAMPs in GBCCL

For the last 15 years, we have been developing a DC-based immunotherapy that improves the long-term survival of patients with advanced melanoma [11]. In our approach, a lysate derived from a mix of three heat shock-conditioned allogeneic melanoma cells (Mel1, Mel2, and Mel3), named TRIMEL, has been used as a source of both TAAs and DAMPs. Heat shock-induced DAMPs, particularly plasma membrane translocated eCRT and released HMGB1, mediate an optimal antigen presenting cell (APC) maturation and antigen cross-presentation, providing a unique strategy to obtain efficient tumor antigen-presenting cells with a mature DC-like phenotype [13].

**Fig. 2:**
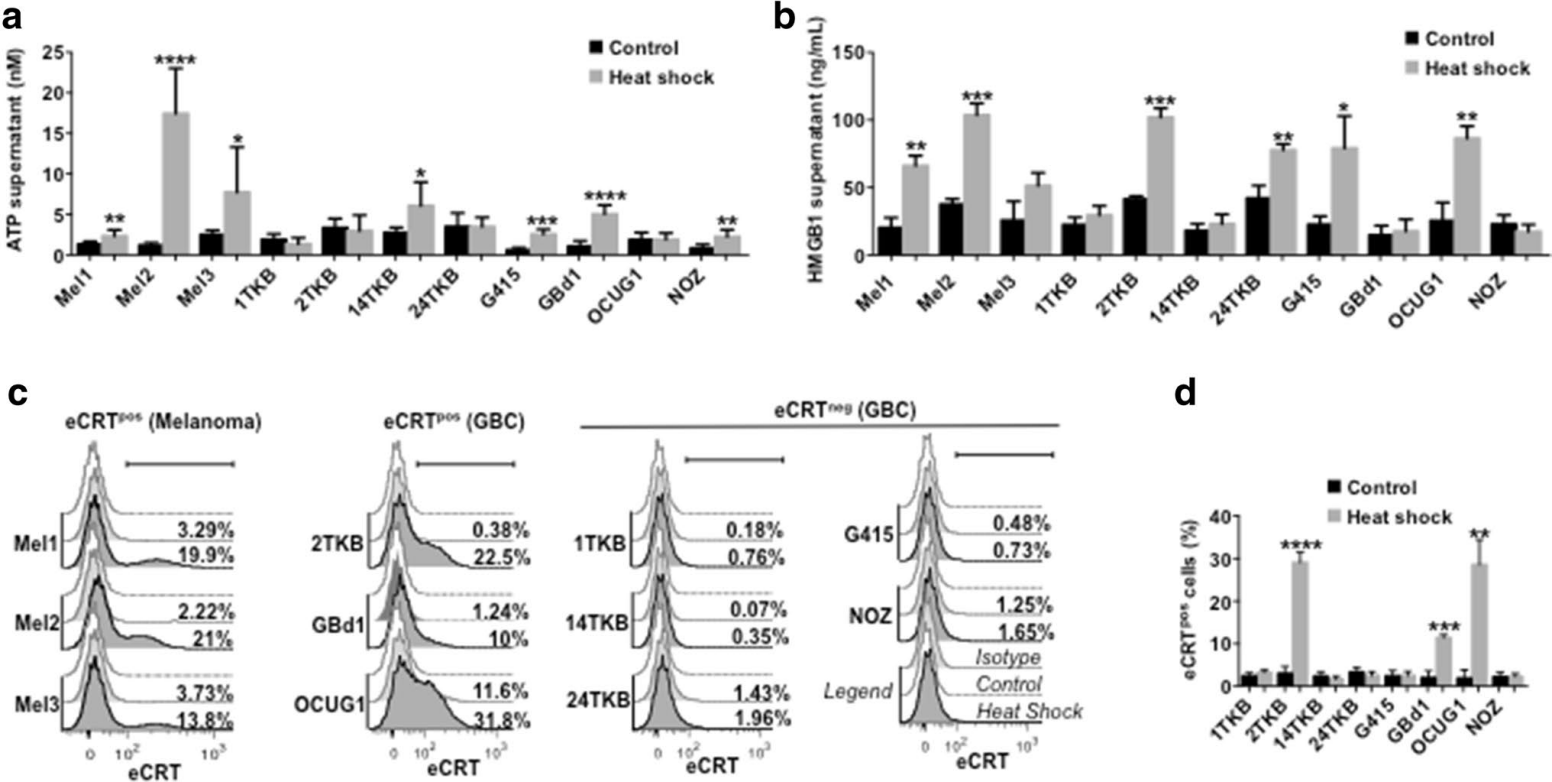
Heat shock conditioning induces DAMP production in GBCCL. The levels of ATP (**a**) or HMGB1 (**b**) were evaluated in the supernatants from heat shock-treated or control cells. **c** Representative histograms showing the extracellular expression levels of translocated calreticulin (eCRT) in heat shock-treated (dark grey) or control (light grey) melanoma and GBC cells. White histograms indicate isotype control staining. The percentage of eCRT positive (eCRT^pos^) for each condition is shown. **d** Statistical analysis of eCRT translocation induced by heat shock in GBCCL. Bars represent averages and standard deviations of three (**b**–**d**) or five-seven (**a**) measurements of three independent experiments. *p < 0.05; **p < 0.01; ***p < 0.001; ****p < 0.0001

Here, we evaluated the production of three common DAMPs (released HMGB1 and ATP, and translocated eCRT) in GBCCL subjected to heat shock. Heat shock treatment induced HMGB1 and ATP release in four of the eight cell lines evaluated (14TKB, G415, GBd1 and NOZ for ATP; and 2TKB, 24TKB, G415 and OCUG1 for HMGB1) (Fig. 2a, b). Additionally, three GBCCL translocated eCRT to the plasma membrane in response to heat shock (2TKB, GBd1 and OCUG1) (Fig. 2c, d). The levels of heat shock-induced DAMPs in GBCCL were similar that those induced in the melanoma cell lines Mel1, Mel2 and Mel3, which were used as positive controls.

### Heat shock-conditioned GBCCL lysate mixtures, but not lysates from individual cell lines, induce differentiation of activated monocytes into mature DCs

**Fig. 3:**
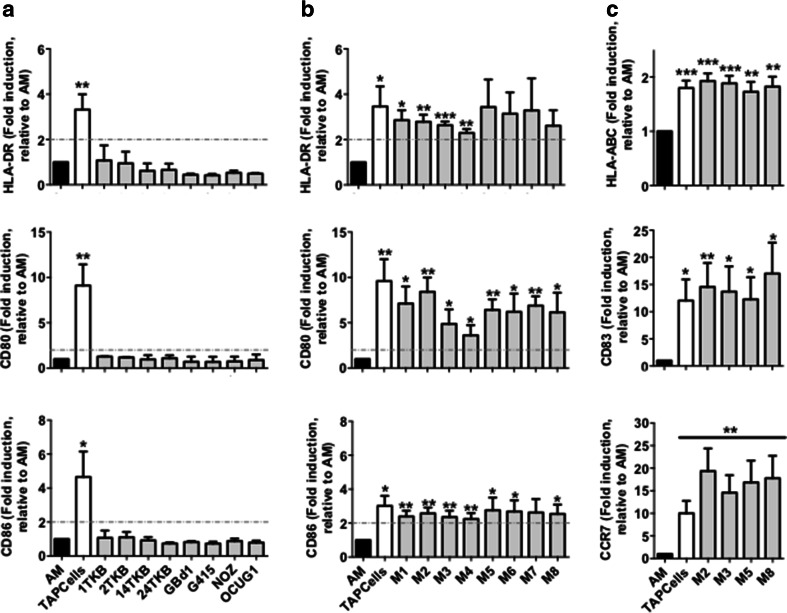
Heat shock-conditioned GBCCL lysate mixtures, but not lysates from individual cell lines, induce differentiation of activated monocytes into mature DCs. Surface expression of HLA-DR, CD80, CD86 (**a, b**), and HLA-ABC, CD83, and CCR7 (**c**) were evaluated by flow cytometry on activated monocytes (AM) incubated or not for 24 h with 100 µg/mL of heat shock-conditioned tumor lysates generated from individual GBCCL (**a**) or mixtures (M1-M8) of three different GBCCL (**b, c**). Bars represent the average and SD of the fold induction of the integrated MFI (iMFI: % positive cells × geoMFI of positive cells) for each marker relative to AM from at least three independent experiments. Evaluated cell lysates mix were made as follows: M1 (24TKB + GBd1 + G415); M2 (2TKB + 24TKB + GBd1); M3 (1TKB + 2TKB + 24TKB); M4 (OCUG1 + GBd1 + G415); M5 (2TKB + G415 + OCUG1); M6 (NOZ + OCUG 1 + G415); M7 (1TKB + 14TKB + 24TKB); and M8 (24TKB + OCUG1 + G415). *p < 0.05; **p < 0.01; ***p < 0.001

As previously reported [13, 20], the addition of TRIMEL to IL-4/GM-CSF-activated monocytes (AM) mediated up to threefold induction of surface markers associated with DC maturation such as HLA-DR, CD80 and CD86 (Fig. 3a). However, heat shock-conditioned lysates prepared from each of the GBCCL did not induce a significant increase in the expression of these markers in stimulated AM (Fig. 3a). Given that the combination of different molecular factors present in each cell line may synergistically contribute to the DC stimulatory activity of the conditioned lysates as in TRIMEL, we produced eight different heat shock-conditioned lysates (M1-M8) combining three different GBCCL in each lysate. The cell lines composing each mixture lysate are described in the Methods section and were chosen according to their tumor antigen expression and presence of heat shock-inducible DAMPs. Unlike individual cell lysates, GBCCL mixture lysates significantly induced the expression of CD80, CD86 and HLA-DR in DCs (Fig. 3b). We extended the analysis to three additional markers: HLA-ABC, CD83 and CCR7 for four of these mixtures of GBCCL lysates: M2, M3, M5 and M8 (Fig. 3c), which were selected considering the antigen expression and DAMP production of the composing cells and the DC stimulatory activity of the lysate. The addition of M2, M3, M5, M8 or TRIMEL lysates mediated the induction of these maturation markers in DCs (Fig. 3c).

### DCs matured with GBCCL lysates induced the activation of allogeneic CD4^+^ and CD8^+^ T cells

**Fig. 4:**
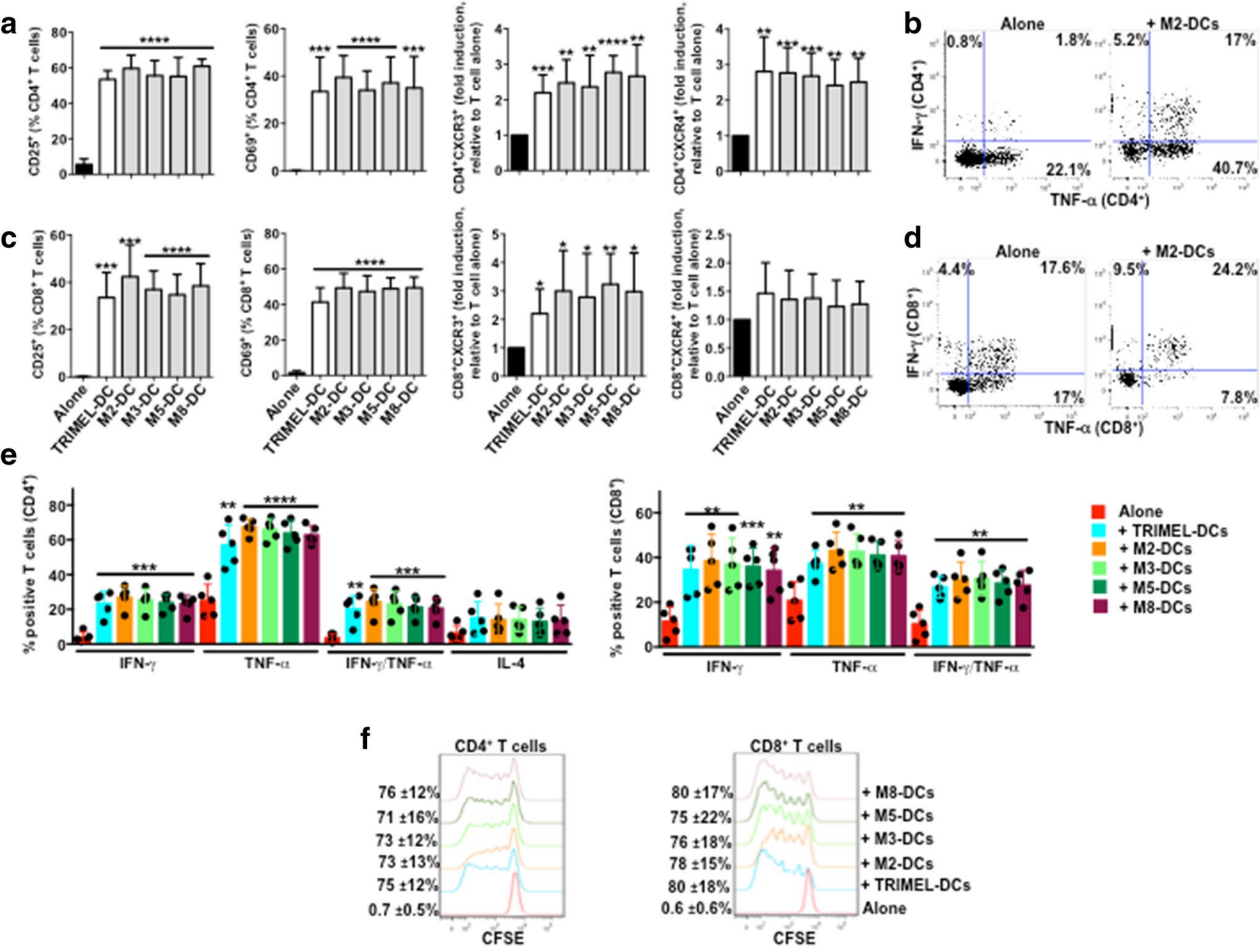
Activation of allogeneic T cells by monocyte-derived DCs matured with different heat shock-conditioned GBC lysates. Purified CD3^+^ T cells were co-cultured for 5 days with allogeneic TRIMEL-, M2-, M3-, M5-, M8-DCs or without DCs. The surface expression of CD25, CD69, CXCR3 and CXCR4 (**a**), the intracellular levels of IFN-γ, TNF-α and IL-4 (**b**–**d**), and proliferation (**e**) were evaluated in the CD4^+^ and CD8^+^ T cells populations by flow cytometry. **a, d** Bars represent the average and SD from five independent experiments of the % of T cells positive for each marker, with the exception of CXCR3 and CXCR4 data that are shown as fold induction of the MFI relative to unstimulated T cells. Representative dot plots of IFN-γ and TNF-α production in allogeneic CD4^+^ (**b**) and CD8^+^ (**c**) T cells co-cultured with M2-DCs. **e** The percentage and SD of proliferating T cells are showed on the left of each histograms. Evaluated cell lysates mix were made as follows: M2 (2TKB + 24TKB + GBd1); M3 (1TKB + 2TKB + 24TKB); M5 (2TKB + G415 + OCUG1); and M8 (24TKB + OCUG1 + G415). *p < 0.05; **p < 0.01; ***p < 0.001; ****p < 0.0001 (comparison versus unstimulated T cells)

To determine GBCCL lysates with major potential to induce mature DCs, we investigated the capacity of DCs matured with the GBCCL lysates M2, M3, M5 and M8 (named M2-DCs, M3-DCs, M5-DCs and M8-DCs, respectively) or with TRIMEL (TRIMEL-DCs, as a positive control) to activate allogeneic T cells. After 5 days of DC/T cell co-cultures, we evaluated the surface expression of the lymphocyte activation markers CD25 and CD69 and the chemokine receptors CXCR3 and CXCR4 on CD4^+^ and CD8^+^ T cells. All the DCs tested induced increased levels of CD25 and CD69 in both subsets (Fig. 4a). Moreover, all DCs were able to induce the expression of both receptors CXCR3 and CXCR4 in CD4^+^ T cells (Fig. 4a) whereas only the chemokine receptor CXCR3 was induced in CD8^+^ T cells co-cultured with all the DC types (Fig. 4a). Additionally, our results demonstrated that both CD4^+^ and CD8^+^ T cells co-cultured with allogeneic DCs loaded with GBCCL heat shock-conditioned lysates expressed high levels of the Th1 cytokines IFN-γ and TNF-α, whereas co-cultured CD8^+^ but not CD4^+^ T cells expressed the Th2 polarizing cytokine IL-4 (Fig. 4b–d). Finally, all the DCs evaluated induced the proliferation of both CD4^+^ and CD8^+^ allogeneic T cells (Fig. 4e).

Based on these results, we selected M2-DCs (loaded with heat shock-conditioned lysate from 2TKB, 24TKB and GBd1 GBCCL) for further experiments. The cell lines composing the M2 lysate were adenocarcinoma cell lines (the most common histology of GBC) and combined they could provide a complete panel of TAAs and DAMPs (Figs. 1c, 2).

### T cells activated by autologous M2-DCs recognize HLA-A2-matched GBCCL

**Fig. 5:**
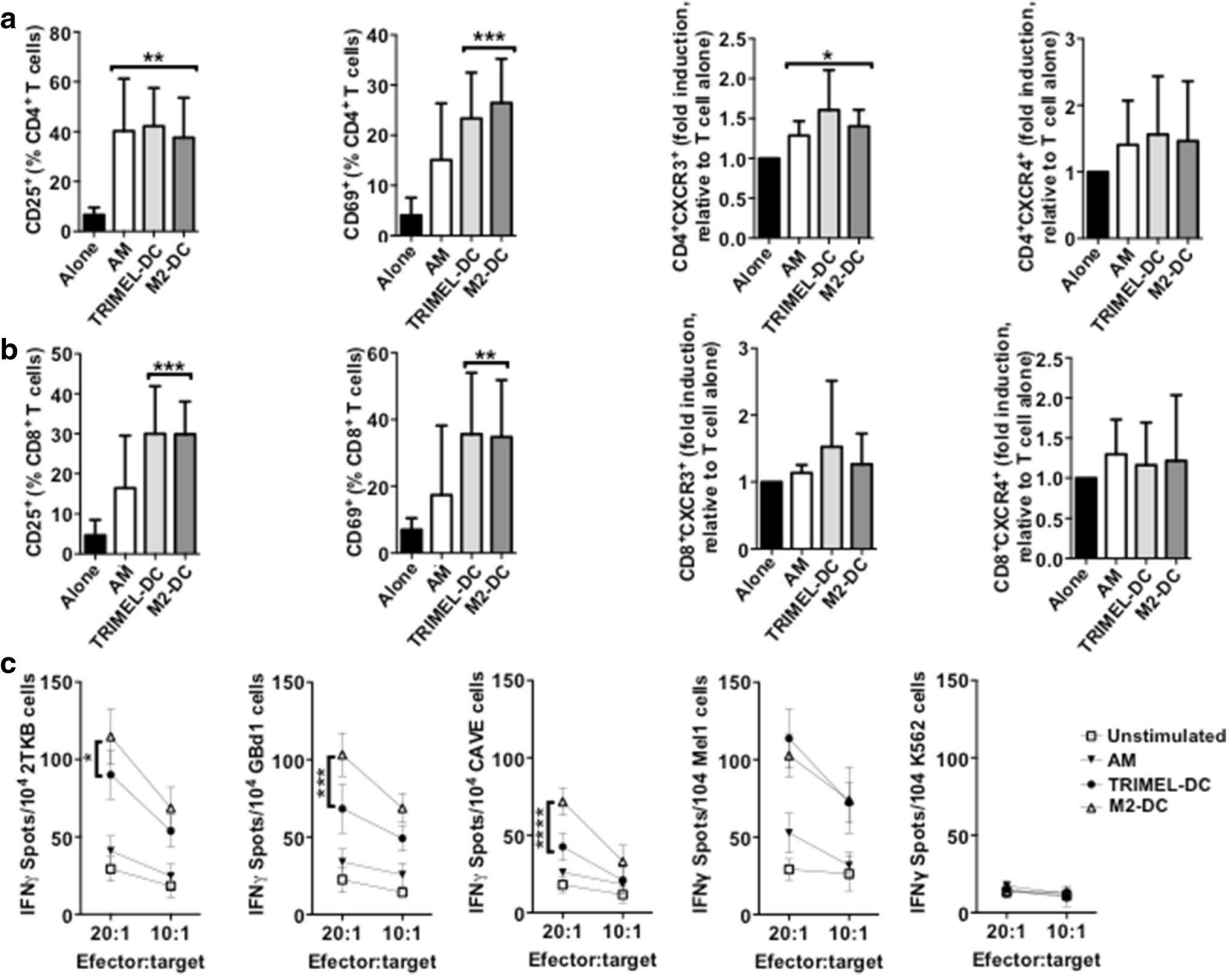
T cells activated by autologous monocyte-derived DCs loaded with a heat shock conditioned GBC lysate recognize HLA-A2-matched GBCCL. **a**–**c** Purified CD3^+^ T cells were co-cultured for 14 days with autologous HLA-A2^+^ AM, TRIMEL-DCs, M2-DCs or cultured alone. The surface expression of CD25, CD69, CXCR3 and CXCR4 (**a, b**) were evaluated in the CD4^+^ (**a**) and CD8^+^ (**b**) T cells populations by flow cytometry. Bars represent the average and SD from at least three independent experiments of the % of T cells positive for each marker, with the exception of CXCR3 and CXCR4 data that are shown as fold induction of the MFI relative to unstimulated T cells. *p < 0.05; **p < 0.01; ***p < 0.001 (comparison versus unstimulated T cells). **c** Sorted CD8^+^ T cells were challenged for 16 h with the HLA-A2^+^ GBCCL 2TKB, GBd1, CAVE, the melanoma cell line Mel1 or K562 cells. IFN-γ release was measured by ELISPOT at different effector:target ratios as indicated. Data represent the average and SD of at least three independent experiments. *p < 0.05; ***p < 0.001; ****p < 0.0001 (comparison M2-DC versus TRIMEL-DCs stimulated T cells). M2 refer to the mixture made from three different GBCCL

Given that the heat shock-conditioned M2 lysate potentially contains a large number of GBC tumor-antigenic epitopes for priming T cell responses, we investigated whether CD8^+^ tumor-specific IFN-γ-secreting T cells were also being elicited in vitro by autologous HLA-A2^+^ M2-DCs. First we observed that M2-DCs were able to activate autologous CD4^+^ and CD8^+^ T cells, measured by the percentage of T cells expressing CD25 and CD69 after 14 days of co-culture (Fig. 5a, b). Then, CD8^+^ T cells were isolated after co-culture by cell-sorting and challenged with two HLA-A2^+^ GBCCL present in the M2 lysate (2TKB and GBd1), a HLA-A2^+^ GBCCL that was not included in the M2 lysate (CAVE), a HLA-A2^+^ melanoma cell line (Mel1), or with K562 cells as a negative control. After challenging with 2TKB, GBd1 or CAVE cells, M2-DC-activated CD8^+^ T cells released significantly higher levels of IFN-γ than CD8^+^ T cells unstimulated or co-cultured with AM or TRIMEL-DCs (Fig. 5c). The NK cell-sensitive cell line K562 did not induce IFN-γ release by the activated CD8^+^ T cells. Additionally, we observed that there was an important cross-recognition of melanoma cells by T cells activated with M2-DCs (Fig. 5c). Similarly, T cells activated with TRIMEL-DCs were able to cross-recognize GBC cells, which may be indicative of shared antigens between both kinds of tumor cells.

## Discussion

Exploration of new active immunotherapies as complements to the relatively recent approaches grounded on blockade of immune checkpoint molecules, such as cytotoxic T-lymphocyte antigen 4 (CTLA-4), programmed death (PD)-1 and PD-ligand-1 (PD-L1), may constitute a feasible possibility for improvement of clinical response rates. Particularly, DC-based cancer vaccines again become an interesting alternative because of their relative effectiveness in activating cell-mediated immune responses and lack of severe side effects in patients [21]. In this context, whole tumor cell lysates are excellent sources for the delivery of a wide range of TAAs that will generate MHC class I/II T cell epitopes for inducing the activation of CD4^+^ T helper and CD8^+^ cytotoxic T cells simultaneously, and therefore, a more integral immune response.

One method to determine the potential usefulness of DC-based immunotherapy in GBC patients is to explore the immunogenicity of GBC tumors by measuring the impact of T cell subpopulation infiltration at tumor sites and to correlate this with the overall survival of patients. Tumor-infiltrating immune cells constitute an accepted manifestation of the host immune response against cancer. Likewise, a relationship between tumor-infiltrating immune cells and GBC prognosis has been suggested. In fact, recent published data from our and other groups showed that CD8^+^ T cell infiltration at different disease stages correlates with improved survival of GBC patients [22–24]. In one study, in which 45 tumor samples from GBC patients and 65 benign gallbladder tissues were examined, increased frequencies of CD4^+^, CD8^+^ T cells and DCs were observed in GBC samples, which significantly correlated with prolonged patient survival [23]. In a more recent study, Oguro and coworkers [25] analyzed 211 GBC samples and found that a lower density of tumor-infiltrating CD8^+^ cells and higher ratios between Foxp3^+^/CD4^+^, B and T lymphocyte attenuator/CD8^+^, and casitas-B-lineage lymphoma protein-b/CD8^+^ were significantly associated with shorter overall survival in GBC patients. Moreover, in a cohort of 80 Chilean GBC patients, we observed that a greater infiltration of CD8^+^ T cells in cancer tissue was associated with a favorable prognostic biomarker for both early and advanced stage patients [24]. Altogether, these observations strongly indicate that a natural host CD8^+^ T cell-mediated immune response against GBC increases patient survival. These findings encourage the design and development of adjuvant immunotherapeutic approaches against GBC.

The aforementioned GBC T cell infiltration might be orchestrated by the chemokine receptor CXCR4, given that its ligand, C-X-C motif ligand-12 (CXCL12), is frequently overexpressed in GBC [26]. Likewise, the expression of CXCR3 by lymphocytes can mediate its migration to GBC tumor beds [27]. These data suggest that the induction of these chemokine receptors in T cells by therapeutic DCs would be beneficial for the DC-mediated anti-tumor responses in vaccinated patients.

The potential use of immunotherapeutic approaches for GBC has only recently become a subject of intensive investigation. In fact, current immunotherapies against GBC have been focused on the use of peptide-based vaccines or peptide-loaded DCs [21, 28]. These strategies have shown modest clinical improvements, likely due to induced tolerance by dominant single tumor peptides or by the selection of antigen loss variants in established tumors. In contrast, a study where DC loaded with autologous tumor cell lysates combined with activated T cell transfer were used as an adjuvant treatment in operated patients with advanced intrahepatic cholangiocarcinoma, reported improved post-operative progression-free and overall survival compared to patients receiving surgery alone [29].

The optimal delivery of tumor antigens is one of the most important factors for the success of DC-based anti-cancer vaccines. With this in mind, lysates from allogeneic tumor cells, whole tumor cells, tumor mRNA, and antigenic peptides, have all been tested as tumor vaccines. Autologous whole tumor antigens offer an unparalleled advantage as it allows DCs to process and present a broad range of TAAs to stimulate strong, polyclonal and long-term memory CD4^+^ and CD8^+^ T cell responses, potentially preventing tumor immune escape. Moreover, this strategy is suitable for all cancer patients regardless of their HLA haplotype. However, not all cancer patients have surgically removable tumors, and therefore, a useful and promising alternative is the preparation of allogeneic cancer cell lysates that have demonstrated to provide a standardized applicable source of tumor-specific antigens in patients with non-resectable tumors [30]. Importantly, the method used for inducing cell death or protein chemical modifications during whole tumor lysate preparation could impact the immunogenicity and efficacy of the therapy (Table 1). Current immunogenic treatment modalities used for pre-conditioning tumor cell lysates include ultraviolet irradiation, oxidation-inducing modalities and heat shock treatments [31]. In the present study, we generated heat shock-conditioned tumor lysate for GBC (M2), which have some important characteristics that suggest its potential as an antigen source for DC vaccines: (1) it contains a broad panel of TAAs, also expressed in tumors from GBC patients, (2) it includes different molecules that could act as DAMPs (released HMGB1, ATP and eCRT), (3) it promotes a rapid and efficient differentiation of monocytes to mature DCs, and (4) DCs generated with this lysate are able to induce the activation of T cells that specifically recognize tumor cells.

**Table 1:** Current protocols used for whole tumor lysate preparations in immunogenic DC vaccines

Strategy	TAAs/neo-Ags	Whole tumor cells	Whole tumor cells + cytokines	Tumor lysates	Conditioned tumor lysates
Mechanism					
Advantages	Specific immuno-dominant Ags NeoAgs-reactive T cells are not affected by central tolerance Personalized	Broad range of TAAs Haplotype-independent Personalized repertoire of TAAs (autologous)	Broad range of TAAs Haplotype-independent Cytokines induce recruitment, activation or proliferation of immune cells	Broad range of TAAs Haplotype-independent Standardized preparation and feasibility of mass production	Broad range of TAAs Haplotype-independent Presence of maturation stimuli for DCs Feasibility of mass and standard production
Disadvantages	Lack of Ag diversity and DC maturation stimuli Immune evasion of tumor cells lacking specific-Ag expression	Lack of DC maturation stimuli Generation of immuno-suppressive molecules Limited to patients with surgically accessible tumors (autologous) Lack of patient-specific Ags (allogeneic)	Lack of DC maturation stimuli Irradiation could generate immuno-suppressive molecules Lack of patient-specific Ags (allogeneic)	Lack of DCs maturation stimuli Possible presence of immunoregulatory molecules from tumor cells (i.e., IL-10, TGF-β) Lack of patient-specific Ags (allogeneic)	Possible presence of immunoregulatory molecules from the tumor cells (i.e., IL-10, TGF-β) Lack of patient-specific Ags (allogeneic)
Clinical Outcomes (patients stage; trial´s phase; type of tumor)	Increased neoAg-specific T cells and detection of HLA class I-restricted neo-Ags (III; phase I; melanoma) [32] 4/6 patients without recurrence after 25 months post-vaccination (III/IV; phase I; melanoma) [33] Adoptive cell transfer of mutation-specific Th1 cells led to tumor regression and stabilization of disease (IV; phase I; cholangiocarcinoma) [34]	Increased overall survival (OS) (39% vs 20%) (IV; phase II; melanoma) [35] Increased median disease free survival (MDFS) from 7 to 20 months (III; phase II; melanoma) [36] Mean OS of 21.9 months. Positive correlation between antibody titer and OS (IV; phase II; colon) [37] Patients developed immune reactions against the tumor. One patient with complete tumor regression (III; phase I; ovarian) [38]	Mean disease free interval of 28.8 months (IV; phase I; breast) [39] Activation of the immune system but no tumor regression (III/IV; phase I/II; lung) [40] Median OS of 34.9 months (high dose group) compared to 24 months (low dose group) (IV; phase I/II; prostate) [41] Low toxicity and generation of cellular immune response (II/III/IV; phase I; melanoma) [42] 50% of patients with stable disease and a median OS of 40 months compared with historic median OS of 10 months (IV; Phase I; renal) [43]	Median OS of 11.5 months (compared with historic median OS of 9.6 months) and 1- and 2-year survival rates of 50 and 27%, respectively. 7/22 patients with stable disease (IV; phase II; mesothelioma) [44] Survival benefit in patients expressing the Ags HLA-A2 and HLA-C3 (38% of clinical response rate versus 7% in patients without expression of the Ags) (III/IV; phase II/III; melanoma) [45] 2/14 patients experienced minor or partial responses (tumor size decrease) (IV; phase I; melanoma) [46]	2 out of 5 patients experienced progression-free survival intervals of 36 and 44 months (II/IV; phase I; ovarian) [47] Stage IV DTH+ patients showed a median OS of 33 months compared with the 11 months from DTH− patients. All stage III patients were DTH+ and remained tumor-free for a median follow-up of 48 months (III/IV; phase II; melanoma) [11]

In general, in vivo tumor antigen presentation by immunotherapeutic DCs might drive the development of tumor-specific adaptive immune responses, whereas cytotoxic CD8^+^ T cells recognize and attack tumor cells through recognition of TAA peptides associated to MHC class I. Therefore, T-cell cytotoxicity depends on MHC class I expression on tumor cell surface. It has been frequently observed that tumor cells lost MHC class I expression, and therefore, the efficacy of DC-mediated immunotherapies may be reduced. In line with this hypothesis, it has been shown that reduced MHC class I expression in biliary tract cancers, including GBC, was linked to shortened overall patient survival [48]. However, in the majority of cases the loss of MHC class I is partial, affecting only some isotypes, and thus an important portion of cancer patients could benefit from DC-mediated immunotherapy. Moreover, it is very important to incorporate strategies to recover MHC class I expression in tumors to improve immunotherapy effect [49]. In conclusion, we propose that GBC cell lysate-loaded DCs may be considered for future immunotherapy approaches alone or in combination with currently used immune checkpoint molecule-blocking therapies.

## Abbreviations

AM: Activated monocytes
BAGE: B melanoma antigen
CA19-9: Cancer antigen 19-9
CCR7: C-C chemokine receptor type 7
CEA: Carcinoembryonic antigen
CXCR: C-X-C motif chemokine receptor
DAMPs: Damage associated molecular patterns
DC: Dendritic cell
eCRT: Extracellular (or exo) calreticulin
FBS: Fetal bovine serum
GAGE: G antigen
GBC: Gallbladder cancer
GBCCL: Gallbladder cancer cell line
HLA: Human leukocyte antigen
HMGB1: High mobility group box-1
IFN-γ: Interferon gamma
iMFI: Integrated mean fluorescence intensity
MAGE: Melanoma-associated antigen
M2-DCs: Dendritic cells matured with M2 lysate
M3-DCs: Dendritic cells matured with M3 lysate
M5-DCs: Dendritic cells matured with M5 lysate
M8-DCs: Dendritic cells matured with M8 lysate
mAbs: Monoclonal antibodies
MHC: Major histocompatibility complex
MUC-1: Mucin-1
PBMC: Peripheral blood mononuclear cells
rhGM-CSF: Recombinant human granulocyte–macrophage colony-stimulating factor
rhIL: Recombinant human interleukin
TAAs: Tumor associated antigens
Th1: Type 1 T helper cells
Th2: Type 2 T helper cells
TNF-α: Tumor necrosis factorα
TRIMEL-DCs: Dendritic cells matured with TRIMEL
